# Effects of high-intensity ultrasound and oil type on the Maillard reaction of d-glucose and glycine in oil-in-water systems

**DOI:** 10.1038/s41538-017-0010-4

**Published:** 2018-01-22

**Authors:** Hang Yu, Yi-Xin Seow, Peter K. C. Ong, Weibiao Zhou

**Affiliations:** 1 Food Science & Technology Programme, c/o Department of Chemistry, National University of Singapore, 3 Science Drive 3, Singapore, 117543 Singapore; 2 KH Roberts Pte. Ltd., 19 Wan Lee Rd, Singapore, 627948 Singapore; 3 National University of Singapore (Suzhou) Research Institute, 377 Linquan Street, Suzhou Industrial Park, Jiangsu 215123 People’s Republic of China

**Keywords:** Natural product synthesis, Chemical engineering

## Abstract

This study addresses the effect of high-intensity ultrasonic processing on four oil-in-water systems, using sunflower, peanut, olive and flaxseed oils, respectively, that contained an aqueous d-glucose and glycine Maillard reaction (MR) model system. The MR in the water phase was promoted as observed from higher depletion of reactants and higher amount of MR products (MRPs). A significantly higher amount of pyrazines was generated after ultrasonic processing, particularly in the sunflower and olive oil systems. These promotions were attributed to a well-mixing effect and a localised high temperature and pressure environment generated by the high-intensity ultrasound. However, upon 1 h of ultrasonic processing at 80 °C, a significant increase of oxidation was observed with high peroxide and *p*-anisidine values in the post-processed oils; meanwhile, the amount of unsaturated fatty acids decreased as well. As a result, some off-flavours were also detected in the post-processed oils, which affected the overall flavour profile of the MR systems.

## Food processing: ultrasound betters flavour off sugar and amino acid

Ultrasound generates more flavour species in oil-in-water mixtures containing sugar and amino acid than heating, but meanwhile degrades oils. Weibiao Zhou at National University of Singapore and collaborators in China studied the effect of high-intensity ultrasonic processing on model food systems with sugar and amino acid in mixtures of water and different oils. The researchers found that high-intensity ultrasound can speed up the Maillard reaction, a chemical reaction that yields various flavour compounds from amino acids and reducing sugars, because it can induce a temporary condition of high temperature and high pressure and promote the mixing between water and oil. On the other hand, ultrasound is responsible for increased oxidation of unsaturated oils, leading to off-flavours and loss of nutrients. These results may help improve ultrasound-assisted food processing techniques.

## Introduction

During food processing with heat, amino acids, peptides and some proteins are capable of reacting with reducing sugars to generate various coloured and odour-active compounds; this reaction has been named as Maillard reaction (MR). Simultaneously, fats/oils in a typical food system may also undergo degradation due to oxidation and thermal reaction during food processing. Therefore, it is necessary to study the MR in the presence of oils to elucidate the relationship between the MR and lipid degradation in food systems as a result of processing.^1^

Previous studies already revealed that the final stage of MR is responsible for the generation of coloured and volatile MR products (MRPs).^2,3^ Melanoidins refer to a group of coloured MRPs. If melanoidins are produced mainly in the final stage, they will result in food browning. Some flavour compounds generated from the MR contribute to a pleasant smell, e.g., pyrazines, alkylpyrazines, furans, pyrroles, etc. Hence, an increased amount of desired flavour compounds via introducing emerging technologies is recommended. Recent studies showed that a number of MRs in model systems were promoted by high-intensity ultrasound in sample solutions.^4,5^ Yu et al.^6^ reported that the generation of flavour compounds was promoted by ultrasound with an intensity of 11.90 W cm^−2^ in a MR model system of d-glucose and l-serine. Studies of xylose–lysine, xylose–cysteine and glucose–methionine model systems also supported such a finding.^7–9^ Therefore, high-intensity ultrasonic processing is a promising technology to promote the MRs. Different types of oil in oil-in-water MR systems would also impact on final flavour profile. For example, Negroni et al.^10^ studied xylose–lysine and glucose–lysine MR model systems in the presence of three oils, i.e., olive, canola and sunflower oils, and indicated that unsubstituted pyrazines were largely formed with olive oil, less with canola oil, and even less with sunflower oil. Therefore, choosing oils with different degree of unsaturation would expect to create different flavour profiles in the same MR model system. In addition, some flavour compounds, such as nonane, 2-pentanone and pyrazines, generated through the MR have good solubility in oil phase; therefore the flavour compounds can easily migrate to the oil phase. Hence, the reaction speed of the MR would be potentially accelerated due to the continuous removal of final MRPs from the water phase where the MR happens the most.

To study an oil-in-water system, changes in the oil phase after processing are of concern. Lipid degradation and oxidation have been reported to be accelerated with the introduction of ultrasound. Chemat et al.^11^ studied the sono-degradation of sunflower oil and found that off-flavour compounds, e.g., limonene and hexanal, were released after sonication of 2 min at 20 kHz of frequency and 150 W of power. Another study focusing on the sono-oxidation of sunflower oil also reported a significant increase in peroxide value (PV) and a decrease in the concentration of unsaturated fatty acids after 30 min of ultrasonic treatment (*f* = 20 kHz and *P* = 150 W).^12^ The localised high temperature and pressure as well as the free radicals generated by cavitation during the ultrasonic processing were responsible for a decline in the degree of unsaturation in the processed oils. Even though high-intensity ultrasound could cause sono-degradation of lipids, it is noticeable that a number of MRPs with strong antioxidant capacity are capable of preventing such degradation in oil-in-water systems.^13^ Vhangani and Van Wyk^13^ determined the reducing power and antioxidant activity of MRPs generated from four lipid-rich MR model systems after thermal processing and observed a decrease in PV and *p*-anisidine value (*p*-AV) of oils, which indicated an inhibition of lipid oxidation brought by MRPs. Among MRPs, melanoidins have a very high antioxidant activity through scavenging free radicals and chelating metals.^14–18^ Besides melanoidins, a number of volatiles, nonvolatile acids and heterocyclic compounds generated by the MR also contribute to different degrees of antioxidant capacity.^19,20^ Elizalde et al.^21^ concluded that volatile MRPs generated in a glucose–glycine MR model system would prolong the induction period of soybean oil oxidation, decreased the rate of oxidation at the propagation stage as well as released less off-flavours, e.g., hexane. Therefore, studying oil properties, i.e., PV, *p*-AV and fatty acid composition, would be essential to study a combined effect of sono-degradation as well as antioxidant capacity brought by MRPs. In addition, measuring acid value (AV) and iodine value (IV) would determine the rancidity and unsaturation of oils before and after processing.

The MR pathway has gradually been revealed, and the thermal processing of d-glucose and glycine MR model system has been well studied. However, there has been no study addressing the effects of high-intensity ultrasound on the MR of d-glucose and glycine and its corresponding oil-in-water MR systems. This research aimed to study changes in both water and oil phases of systems when processed by two different methods, i.e., high-intensity ultrasound and heating.

## Results

### Analysis of raw oils and oil phase

**Fig. 1:**
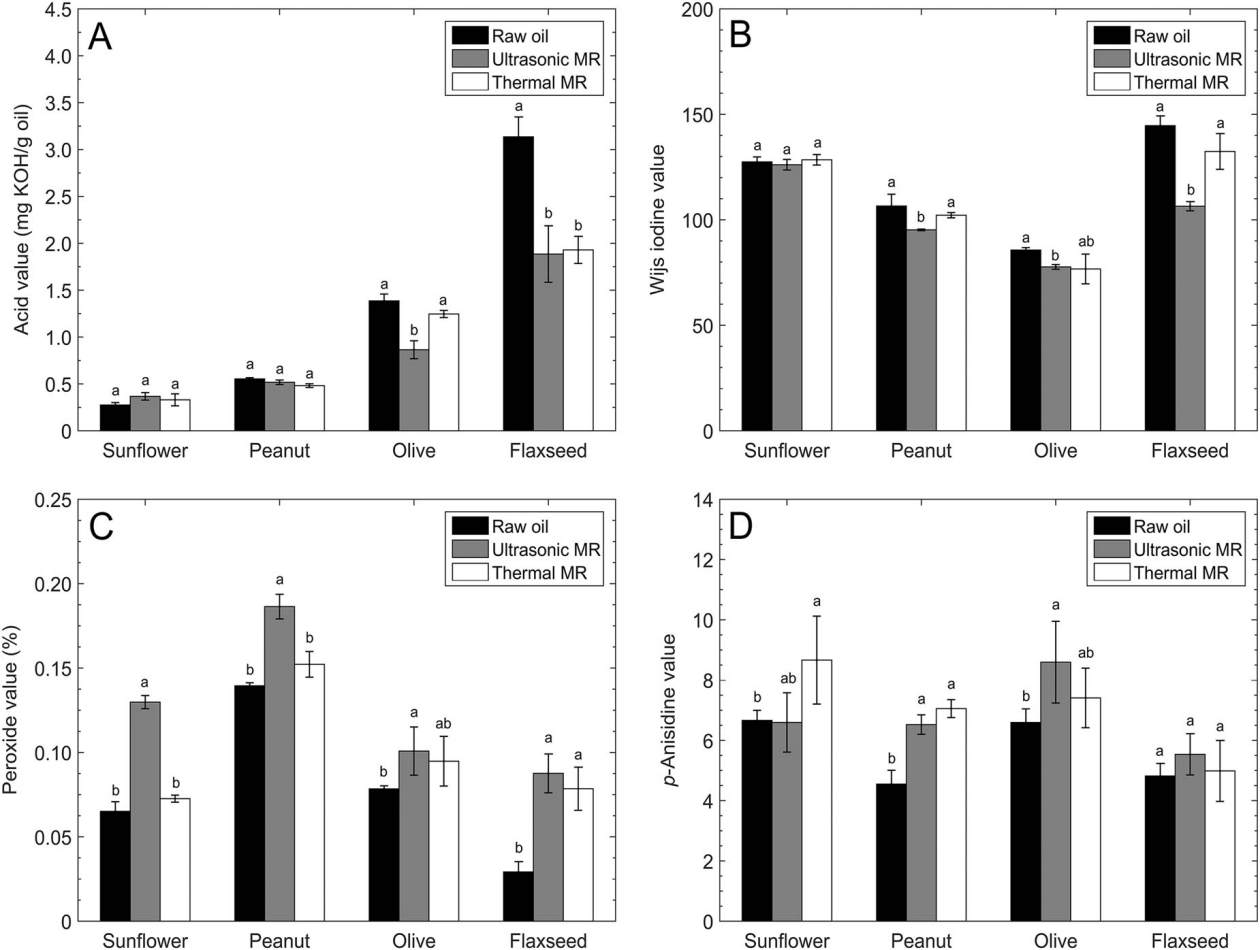
Oil properties of raw oil and oil phase after ultrasonic and thermal processing: **a** acid value, **b** iodine value, **c** peroxide value, and **d** *p*-anisidine value. Significant differences of values within each group are indicated by different letters (*p* < 0.05)

AV is a common measurement of free fatty acid presented in fats or oils. The AV of oil phases after ultrasonic and thermal processing were significantly decreased compared with the corresponding raw oils, except for sunflower and peanut oils which had no significant difference between the raw and processed oils, as shown in Fig. 1a. IV is a commonly used parameter to determine the amount of unsaturation of constituent fatty acids.^22^ The IVs of raw oils and the oil phase of processed samples are shown in Fig. 1b. The raw flaxseed oil had the highest IV with the highest degree of unsaturation, followed by sunflower, peanut and olive oils. Upon ultrasonic processing, the IV of oil phase was significantly decreased compared with the raw oil, for all the types of oil except sunflower oil, for which no significant difference was observed between the raw and processed oils. Thermal processing, however, resulted in a slight decrease of IV without statistical significance for all the four oils. PV and *p*-AV indicate the levels of primary and secondary oxidations, respectively, that have undergone in the tested oils. Compared with the four raw oils, the PV of oil phase samples after ultrasonic processing were all significantly increased, as shown in Fig. 1c. After thermal processing, a trend of increasing PV was observed; however, only the PV of flaxseed oil phase showed a significant increase compared with the raw oil. In general, the oil phase samples after thermal processing had a relatively low PV compared with those after ultrasonic processing. For *p*-AV, thermal processing led to a significant increase except for olive and flaxseed oils, while ultrasonic processing only resulted in an increase for peanut and olive oils, and there was no difference between the ultrasonically and thermally processed samples for all the four types of oil, as shown in Fig. 1d.

Fatty acid compositions of the four raw oils and the oil phases of processed samples are summarised in Table 1. In general, the proportion of both mono-unsaturated fatty acids (MUFA) and poly-unsaturated fatty acids (PUFA) were decreased in the oil phases after ultrasonic and thermal processing compared with the raw oils. Looking at the changes of PUFA, linoleic acid (C18:2) was significantly decreased in the oil phases after ultrasonic and thermal processing in the four oil-in-water systems. The percentage of C18:2 fell from 51.44% in the raw sunflower oil to 51.21 and 51.24% after ultrasonic and thermal processing, respectively, and the decreases were statistically significant. Similarly, statistically significant 0.2 and 0.3% decreases of C18:2 were observed in peanut and flaxseed oils after processing. For olive oil, in particular, almost 1% of linoleic acid was depleted in the ultrasonic-processed sample compared with the raw one. Another PUFA, γ-linolenic acid (C18:3), was also significantly degraded in flaxseed oil after ultrasonic processing. In the thermally processed samples, the percentages of PUFA were decreased compared with the raw oils without statistical significance. As for MUFAs, both ultrasonic and thermal processing caused a 0.5% decrease of oleic acid (C18:1) with statistical significance in peanut and flaxseed oils. A significant decrease of gondoic acid (C20:1) in olive oil was also observed after the two processes.

**Table 1:** Fatty acids composition (%) of raw oils and oil phases of processed samples (*n* = 3)^a^

Fatty acids^b^	Sunflower oil			Peanut oil			Olive oil			Flaxseed oil		
	Raw	Ultrasonic	Thermal	Raw	Ultrasonic	Thermal	Raw	Ultrasonic	Thermal	Raw	Ultrasonic	Thermal
C14:0	–^c^	–	–	–	–	–	0.22 ± 0.02^a^	0.21 ± 0.01^a^	0.25 ± 0.01^a^	–	–	–
C16:0	9.46 ± 0.05^b^	9.72 ± 0.02^a^	9.68 ± 0.09^a^	13.29 ± 0.04^b^	13.62 ± 0.05^a^	13.56 ± 0.05^a^	19.28 ± 0.05^b^	19.97 ± 0.01^a^	19.80 ± 0.01^a^	10.16 ± 0.03^c^	11.32 ± 0.02^a^	10.85 ± 0.07^b^
C16:1	–	–	–	–	–	–	1.76 ± 0.01^a^	1.73 ± 0.02^a^	1.73 ± 0.01^a^	1.53 ± 0.01^a^	1.51 ± 0.01^a^	1.52 ± 0.01^a^
C18:0	3.79 ± 0.04^a^	3.85 ± 0.03^a^	3.78 ± 0.02^a^	3.13 ± 0.01^c^	3.43 ± 0.02^a^	3.28 ± 0.02^b^	2.88 ± 0.03^a^	2.89 ± 0.03^a^	2.85 ± 0.01^a^	8.02 ± 0.02^b^	8.43 ± 0.02^a^	8.37 ± 0.02^a^
C18:1	35.01 ± 0.02^a^	34.93 ± 0.05^a^	35.00 ± 0.05^a^	51.85 ± 0.04^a^	51.37 ± 0.06^c^	51.69 ± 0.01^b^	66.21 ± 0.11^b^	66.75 ± 0.08^a^	66.21 ± 0.11^b^	32.16 ± 0.07^a^	31.51 ± 0.03^b^	31.44 ± 0.06^b^
C18:2	51.44 ± 0.04^a^	51.21 ± 0.06^b^	51.24 ± 0.05^b^	29.02 ± 0.07^a^	28.86 ± 0.02^b^	28.88 ± 0.02^b^	9.08 ± 0.06^a^	8.00 ± 0.09^b^	8.69 ± 0.07^b^	18.52 ± 0.05^a^	18.20 ± 0.03^b^	18.23 ± 0.05^b^
C18:3	–	–	–	–	–	–	–	–	–	29.61 ± 0.01^a^	29.03 ± 0.04^b^	29.59 ± 0.07^a^
C20:0	0.30 ± 0.03^a^	0.29 ± 0.07^a^	0.30 ± 0.02^a^	0.54 ± 0.03^a^	0.57 ± 0.01^a^	0.55 ± 0.02^a^	0.19 ± 0.01^a^	0.21 ± 0.01^a^	0.21 ± 0.01^a^	–	–	–
C20:1	–	–	–	0.69 ± 0.03^a^	0.63 ± 0.01^a^	0.62 ± 0.05^a^	0.38 ± 0.03^a^	0.24 ± 0.01^b^	0.26 ± 0.04^b^	–	–	–
C22:0	–	–	–	1.02 ± 0.05^a^	1.03 ± 0.02^a^	1.01 ± 0.05^a^	–	–	–	–	–	–
C24:0	–	–	–	0.46 ± 0.02^a^	0.49 ± 0.05^a^	0.41 ± 0.02^a^	–	–	–	–	–	–

^a^For every type of oil, significant differences of values within each row are indicated by different letters (*p* < 0.05)

^b^Fatty acids in the first column refer to myristic acid (C14:0), palmitic acid (C16:0), palmitoleic acid (C16:1), stearic acid (C18:0), oleic acid (C18:1), linoleic acid (C18:2), α-linolenic acid (C18:3), arachidic acid (C20:0), gondoic acid (C20:1), behenic acid (C22:0), lignoceric acid (C24:0), respectively

^c^The data presented in this table refer to the percentage of each fatty acid in the total amount of fatty acids in raw oils and the oil phases of processed samples

“–” indicates that the corresponding fatty acid was not detected

### Analysis of water phase

**Fig. 2:**
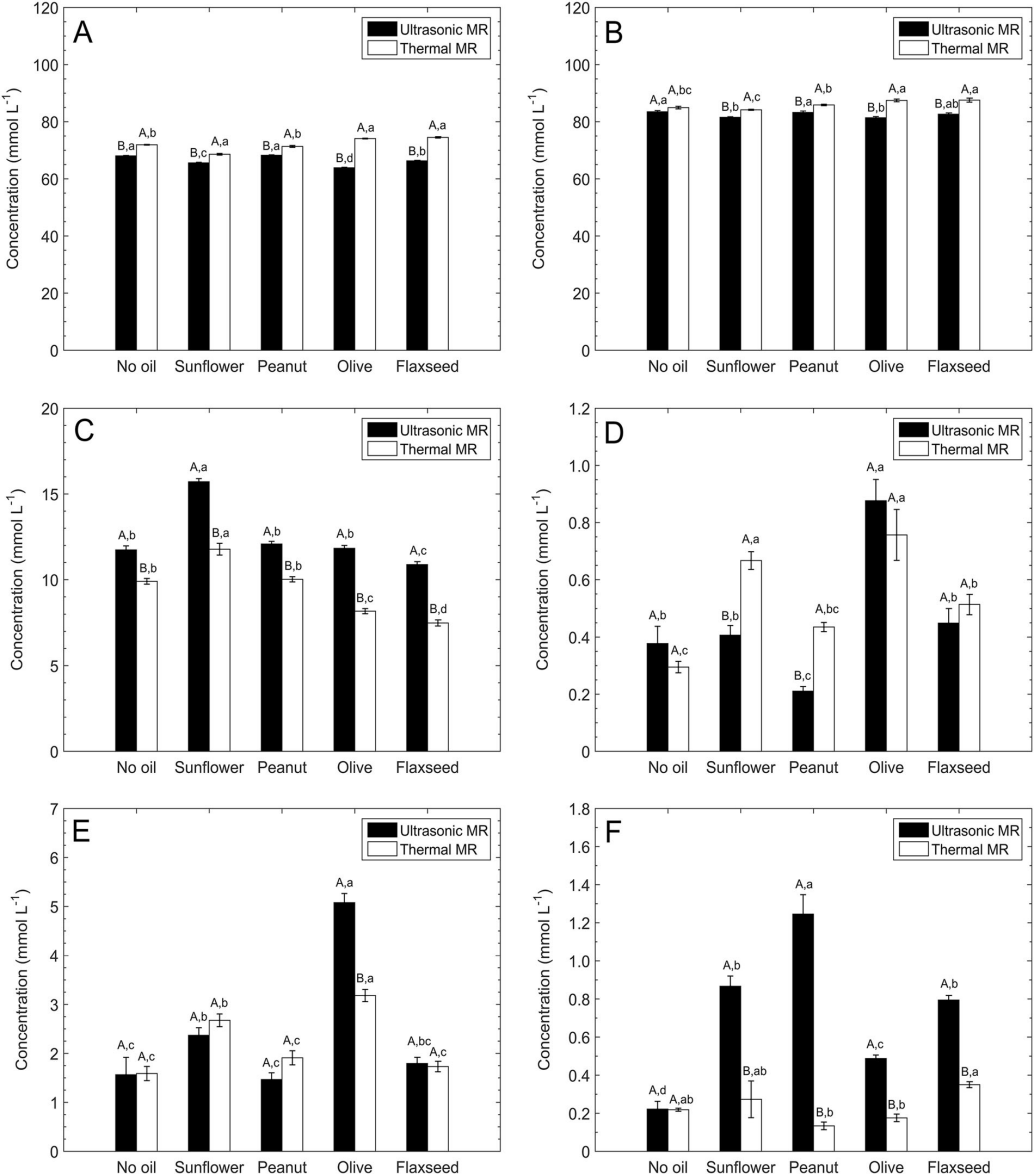
Reactants depleted and MRPs formed in oil-free-MR system and water phase of oil-in-water systems: **a** d-glucose, **b** glycine, **c** fructose, **d** formic acid, **e** acetic acid, and **f** melanoidins. Significant differences of values within each group are indicated by capital letters (*p* < 0.05). Significant differences of values within each of the ultrasonic and thermal MR are indicated by lowercase letters (*p* < 0.05)

As shown in Fig. 2a, for all the MR model systems, the depletion of d-glucose in ultrasonic MR was significantly higher than that in thermal MR. The same trend was observed for the depletion of glycine except for the oil-free-MR system, in which no significance was observed between the two processing methods, as shown in Fig. 2b. For the generation of fructose, which was converted from d-glucose, the higher proportion of d-glucose depleted in any MR model system would generate a corresponding higher concentration of fructose, as shown in Fig. 2c. A significantly lower concentration of reactants and a significantly higher concentration of fructose were detected in the four oil-in-water MR systems after ultrasonic processing compared with their counterparts after thermal processing.

Regarding the generation of organic acids as final MRPs, as shown in Fig. 2d the concentration of formic acid was the same between ultrasonic and thermal processing in the oil-free-MR and oil-in-water MR systems, except for sunflower oil-in-water and peanut oil-in-water MR systems, in which the concentration of formic acid after ultrasonic processing was significantly lower than that after thermal processing. As shown in Fig. 2e, the concentration of acetic acid was the same between ultrasonic and thermal processing in the oil-free-MR and oil-in-water MR systems, except for olive oil-in-water MR system, in which the concentration of acetic acid in ultrasonic processing was significantly higher than that in thermal processing.

Coloured MRPs, melanoidins, were only generated in the final stage of MR. As shown in Fig. 2f, the concentration of melanoidins in the four oil-in-water MR systems by ultrasonic processing was significantly higher than that in the corresponding MR systems by thermal processing.

### Analysis of volatile compounds

All of volatile compounds in the four raw oils as well as oil and water phases after ultrasonic processing were summarised and subsequently classified into five groups, as shown in Table 2. For example, 11 volatile compounds were detected from the four raw oils; however, only four of them remained to be detectable after ultrasonic processing, namely 3-methylbutyraldehyde, haxanal, 2,4-dimethyl heptane and nonanal. The other seven volatile compounds could be degraded after ultrasonic processing; the missing compounds belonged to acids, alcohols and acetates, which had low stability and might be easily destroyed under a high temperature condition. Moreover, there were 13 volatile compounds generated only in the oil phase after ultrasonic processing. Among these volatile compounds, nine of them belonged to alkanes, which contribute to an odour of waxy and fatty. In addition, rancid and metallic odours are related to (E)-2-heptenal. These off-flavours were mainly attributed to the degradation of unsaturated fatty acids. In general, due to the degradation of linoleic acid and sterols, flavour descriptions of fishy, grassy, oxidised, and pungent were described resulting from the generation of the off-flavours after ultrasonic processing of oils.^12^ Five of volatile compounds were detected in both oil and water phases after ultrasonic processing, namely 2-pentanone, 4-methylheptane, nonane, 2,5-dimethylpyrazine and 2,3,5-trimethylpyrazine. These volatile compounds could have been firstly generated by the MR in the water phase and subsequently migrated to the oil phase due to the well-mixing effect of ultrasound. The last two volatile compounds detected in the oil phase, i.e., 2,5-dimethylpyrazine and 2,3,5-trimethylpyrazine, contribute to a pleasant flavour profile of cocoa, coffee, nutty and roasted; 2-pentanone contributes to a flavour profile of sweet, fruity, ethereal and wine. However, the rest two, i.e., 4-methylheptane and nonane, were attributed to an unpleasant odour. Since no sensory evaluation was conducted in this study, it is hard to argue and describe the final odour description of processed oil phases. A number of volatile compounds were only detectable in the water phase after ultrasonic processing, e.g., 3-ethyl-2,5-dimethylpyrazine, 2-methylpyrazine, etc. Possible reasons include a relatively low solubility of the pyrazines in the oil phase compared with that in the water phase, and a relatively low concentration generated in the water phase where the MR happed.

**Table 2:** Summary of volatile compounds detected in samples after 1 h ultrasonic processing at 80 °C

Classification	Retention time	Flavour compounds	Odour descriptions^a^	Types of oil			
				Sunflower	Peanut	Olive	Flaxseed
In raw oils	2.644	Acetic acid	Pungent, sour, vinegar^23^				
	4.719	1-Methylpyrrole	Herbal, smoky, woody^23^	N.D.^b^		N.D.	N.D.
	7.894	Hexanol	Fruity, green, oily	N.D.	N.D.		
	11.201	2-Pentylfuran	Fruity, green, vegetable			N.D.	
	11.399	Decane	Fatty, fresh, waxy				
	11.617	3-Hexenyl acetate	Fresh, green, sweet	N.D.	N.D.		N.D.
	11.789	Hexyl acetate	Fruity, green, sweet	N.D.	N.D.		N.D.
In both raw oils and oil phases after processing	3.841	3-Methylbutyraldehyde	Chocolate, fruity, green			N.D.	N.D.
	6.063	Haxanal	Fatty, fresh, green				
	6.601	2,4-Dimethylheptane	Fatty, fresh, waxy	N.D.		N.D.	N.D.
	14.112	Nonanal	Fatty, fresh, waxy	N.D.	N.D.		N.D.
In oil phases after processing	1.964	3-Methyl-2,5-dihydrofuran	Fruity, green, vegetable		N.D.	N.D.	N.D.
	2.106	Pentane	Fatty, fresh, waxy				N.D.
	4.831	2,3,4-Trimethylpentane	Fatty, fresh, waxy	N.D.		N.D.	N.D.
	6.034	Octane	Fatty, fresh, waxy	N.D.	N.D.		N.D.
	6.387	3-Methylheptane	Fatty, fresh, waxy			N.D.	
	7.711	4-Methyloctane	Fatty, fresh, waxy				
	10.252	(E)-2-Heptenal	Fatty, fresh, green. Rancid, metallic^12^	N.D.	N.D.		N.D.
	10.444	4-Methylnonane	Fatty, fresh, waxy				
	10.775	Tetradecane	Waxy, fatty^23^			N.D.	
	11.196	2-Pentylfuran	Fruity, green, earthy			N.D.	
	11.394	Decane	Fatty, fresh, waxy				
	11.926	2,5-Dimethylnonane	Fatty, fresh, waxy				
	12.981	4-Methyldecane	Fatty, fresh, waxy				N.D.
In both oil and water phases after processing	2.451	2-Pentanone	Sweet, fruity, ethereal, wine^23^	N.D.		N.D.	N.D.
	4.825	4-Methylheptane	Fatty, fresh, waxy		N.D.	N.D.	N.D.
	8.691	Nonane	Gasoline			N.D.	N.D.
	8.974	2,5-Dimethylpyrazine	Cocoa, roasted, nutty				
	11.454	2,3,5-Trimethylpyrazine	Nutty, musty, powdery, cocoa, potato, musty			N.D.	
In water phases after processing	6.078	Ethylbutyrate	Fruity, sweet^23^	N.D.			N.D.
	6.686	2-Methylpyrazine	Nutty, cocoa, roasted, chocolate	N.D.			
	9.223	2,3-Dimethylpyrazine	Nutty, coffee, peanut				
	13.468	3-Ethyl-2,5-dimethylpyrazine	Hazelnut, nutty^23^				
	13.651	2,3,5,6-Tetramethylpyrazine	Musty, nutty, chocolate, coffee, cocoa				
	13.666	5-Ethyl-2,3-dimethylpyrazine	Hazelnut, nutty	N.D.		N.D.	
	15.502	3,5-Diethyl-2-methylpyrazine	Nutty, meaty, vegetable^23^		N.D.		N.D.
	15.517	2-Propyl-3,6-dimethylpyrazine	Hazelnut, nutty	N.D.	N.D.	N.D.	
	16.831	2,4-Dimethylbenzaldehyde	Naphthyl, cherry, bitter-almond^23^			N.D.	N.D.
	23.176	2,4-Di-tert-butylphenol	Phenolic				N.D.

^a^Unless indicated otherwise, odour descriptions were obtained from the website of The Good Scents Company: www.thegoodscentscompany.com

^b^N.D. refers to the compound which was not detectable

It is noteworthy that the flavour profiles of the four oil-in-water MR systems were different from each other. Looking at the MRPs after ultrasonic processing, 2-propyl-3,6-dimethylpyrazine was only generated in the flaxseed oil system. Negroni et al.^10^ conducted a study focusing on flavour compounds generated in a MR system with the addition of olive, canola and sunflower oils. The results indicated that the oils with a higher degree of unsaturation would significantly decrease the amount of unsubstituted pyrazines. Even though no unsubstituted pyrazines was detected in this study, the oils with a high degree of unsaturation promoted the generation of substituted pyrazines. In addition, olive oil had the lowest IV and the relatively low PV and *p*-AV. Thus, three alkanes with fatty, waxy and fresh odours were absent in the olive oil after ultrasonic processing, namely 2,3,4-trimethylpentane, 3-methyloctane and tetradecane.

**Fig. 3:**
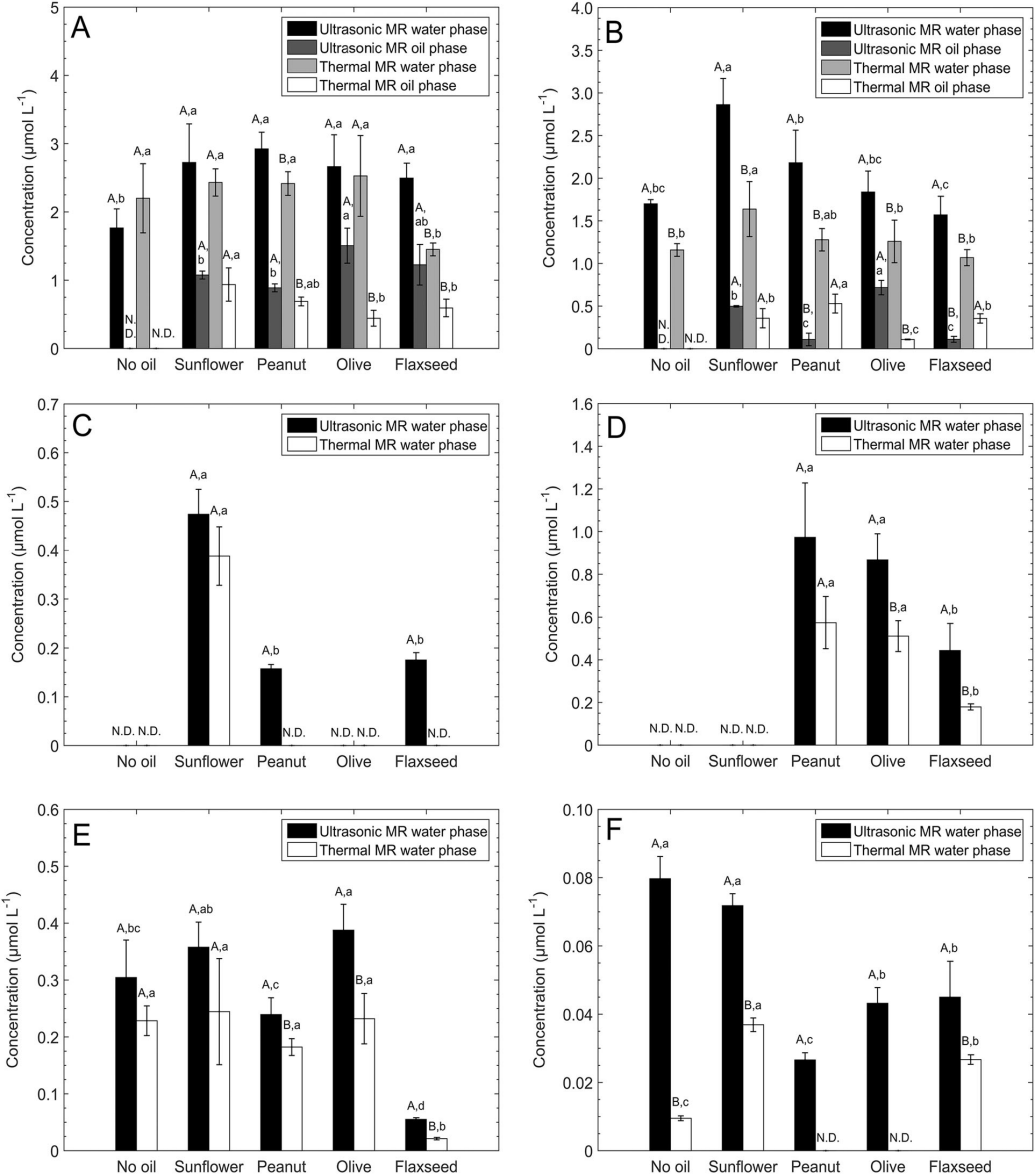
Pyrazines generated in in oil-free-MR system as well as both oil and water phase of oil-in-water systems: **a** 2,5-dimethylpyrazine, **b** 2,3,5-trimethylpyrazine, **c** 2-methylpyrazine, **d** 2,3-dimethylpyrazine, **e** 3-ethyl-2,5-dimethylpyrazine, and **f** 2,3,5,6-tetramethylpyrazine. Significant differences of values within each group are indicated by capital letters (*p* < 0.05). Significant differences of values within each of the ultrasonic and thermal MR are indicated by lowercase letters (*p* < 0.05)

Pyrazines are a group of volatile compounds with strong roast, nutty, cocoa and coffee odours, which are generated by the MR through Strecker degradation.^23,24^ In this study, two pyrazines, 2,5-dimethylpyrazine and 2,3,5-trimethylpyrazine, were mostly generated in the water phase during the MR and partially migrated to the oil phase. Comparing with the oil-free-MR model system, the concentration of 2,5-dimethylpyrazine (odour threshold: 35 ppm in water^25^) in the four oil-in-water MR systems were significantly increased, as shown in Fig. 3a. Considering the total amount of pyrazines detected in both oil and water phases, the system with olive oil generated as high as 4.17 ± 0.72 μmol L^−1^ of 2,5-dimethylpyrazine in total, followed by the system with peanut (3.81 ± 0.30 μmol L^−1^), sunflower (3.80 ± 0.62 μmol L^−1^) and flaxseed (3.72 ± 0.52 μmol L^−1^) oils after 60 min of ultrasonic processing. Similarly, thermal processing was also capable of generating a relatively higher amount of 2,5-dimethylpyrazine in the oil-in-water MR systems compared with the oil-free-thermal MR. Moreover, it is noteworthy that the ratios of 2,5-dimethylpyrazine in water to oil phases were different in the four oil-in-water MR systems. The oil-in-water MR system with sunflower and peanut oils had a relatively low ratio of 2,5-dimehylpyrazine, which was lower than 30%; however, the percentage increased to around 40% in olive and flaxseed oil-in-water MR systems. Such a different distribution could be attributed to a different solubility of 2,5-dimethylpyrazine in the four oils. The generation of 2,3,5-trimethylpyrazine (odour threshold: 9 ppm in water^25^) is shown in Fig. 3b. After ultrasonic processing, the system with sunflower oil generated the highest amount of 2,3,5-trimethylpyrazine (3.36 ± 0.32 μmol L^−1^) accounting both oil and water phases, followed by the system with olive (2.56 ± 0.49 μmol L^−1^), peanut (2.29 ± 0.45 μmol L^−1^) and flaxseed (1.68 ± 0.25 μmol L^−1^) oils. Similarly, the generated amounts of 2,3,5-dimethylpyrazine in the four oil-in-water MR systems after thermal processing were significantly higher than that in oil-free-thermal MR, but still lower than those after ultrasonic processing.

Four other pyrazines were also quantified in our study, namely 2,3-dimethylpyrazine (odour threshold: 2.5 ppm in water^25^), 2-methylpyrazine (odour threshold: 60 ppm in water^25^), 3-ethyl-2,5-dimethylpyrazine (odour threshold: 0.01 ppm in water^25^) and 2,3,5,6-tetramethylpyrazine (odour threshold: 1 ppm in water^25^)^.^ However, all of them were absent in the oil phase. For 2,3-dimethylpyrazine (as shown in Fig. 3c), peanut and flaxseed oil-in-water MR systems were capable of forming it after ultrasonic processing but absent in the same system after thermal processing. Moreover, the highest amount of 2,3-dimethylpyrazine was generated in the sunflower oil-in-water MR system, which was almost 60% higher than those in the oil-in-water MR systems with peanut and flaxseed oils. However, there was lack of 2-methylpyrazine (as shown in Fig. 3d) generated in the oil-free-MR system and the oil-in-water MR system with sunflower oil after both ultrasonic and thermal processing; but it was detected in the other three MR systems. In addition, the lowest concentration of 2-methylpyrazine was formed in the flaxseed oil-in-water MR system compared with the other two MR systems, which was about 50% less than the oil-in-water systems with peanut and olive oils. Similarly, the flaxseed oil-in-water MR system generated the lowest amount of 3-ethyl-2,5-dimethylpyrazine, which was even lower than that in the oil-free-MR system, as shown in Fig. 3e. From Fig. 3f, the concentration of 2,3,5,6-tetramethylpyrazine in the oil-free-MR and sunflower oil-in-water MR model systems was significantly higher than that in the flaxseed and olive oil-in-water MR systems, and the peanut oil-in-water MR system had the lowest concentration among the five systems, which was different from the other pyrazines.

## Discussion

### Analysis of raw oils and oil phase

In the presence of sodium hydroxide in water phase, the triglycerides in raw oils were possibly to be saponified to form glycerol, and released free fatty acid salts, hence, resulting in the decrease of AV. It is noticeable that AV of the samples after ultrasonic and thermal processing were significantly lower than that of the unprocessed oils except for sunflower and peanut oils, in which there was no significant difference between the processed and unprocessed oils. This finding may indicate the saponification of triglycerides in ultrasonic processing. Previous studies have revealed that high-intensity ultrasound was capable of generating a high pressure, shear stress and turbulence.^26^ In addition, cavitation bubbles generated by the high-intensity ultrasound would pyrolyse water molecules that formed a number of free hydroxyl radicals (OH).^27^ Based on these, the reaction between triglycerides and sodium hydroxide was promoted by high-intensity ultrasound, and subsequently a decrease of AVs was observed in the samples after ultrasonic processing.

The decrease of IV after exposure to air and light or after heating and deep-frying processing has been reported, and the mechanism behind the phenomenon is attributed to lipid oxidation, hydrolysis and deterioration of both MUFAs and PUFAs.^28–30^ High-intensity ultrasound characterised by an extremely high, localised temperature and pressure as well as the formation of many free radicals, thus leads to such a degradation of MUFAs and PUFAs in the oils.

As elucidated in our study, oil oxidation occurred in ultrasonic processing. The increased PV of oils after ultrasonic processing may be caused by heating or exposure to active species, e.g., active hydrogen atom (H^+^) due to ultrasonic-induced pyrolyzation of water molecules. Chemat et al.^11^ reported an almost 20% increase of PV in a refined sunflower oil when processed by high power ultrasound; meanwhile, many off-flavour compounds were released due to the oil oxidation. The possible mechanism is as follows: upon formation of sufficient free radicals, the chain reaction on both PUFAs and MUFAs was initiated by the abstraction of H^+^ adjacent to the double bonds and subsequently attacked at these locations resulting in the production of peroxy radicals. Alternative mechanism may be a result of metals naturally contained in the oils and/or metal parts of ultrasonic processor, which results in the formation of oxy radical species in the combination of ultrasonic cavitation.^12^

The degradation of MUFAs and PUFAs in the oils after ultrasonic processing was in accordance with the decrease of IVs except for sunflower oil. The oils after ultrasonic processing had the highest percentage of saturated fatty acid, followed by the oils after thermal processing and the raw oils. The significant increase of C16:0 in the four oils after ultrasonic and thermal processing was observed; however, the percentage of C18:0 was only slightly increased in peanut and flaxseed oils after processing without significance, and no change of C20:0 was observed. It is likely that a number of MUFAs and PUFAs were first degraded to C18:0 and C20:0, and these long-chain FAs might be further decomposed to some shorter-chain FAs during processing, e.g., C14:0 and C16:0. Previous studies have reported similar findings on oils after heating and deep-frying,^29,31–33^ and one of which reported a decrease of both C18:3 and C18:2 content (by around 3%) and an increase of C16:0 in palm and canola oils (by around 2%) after deep-frying at 180 °C.^33^ Such a deep frying process also created a high temperature condition, which had some similarity to the ultrasonic processing in this study due to a momentary but extremely high temperature condition generated by high-intensity ultrasound.

### Analysis of water phase

The difference between ultrasonic and thermal MR on the depletion of reactants and generation of fructose became larger in the oil-in-water MR systems, which indicate a promoted MR at the initial stage in the four oil-in-water MR systems. As discussed in the previous section, flaxseed oil had the highest degree of unsaturation, followed by sunflower, peanut and olive oils. Thus, the promotion of initial stage in MR is possibly linked to the degree of unsaturation of oils. Previous studies have proposed some possible mechanisms of the promotion brought by high-intensity ultrasound,^7^ one of which was a strong agitation and mixing effect generated by ultrasonic cavitation. Due to the high insolubility of oils in the water phase, the mixing effect brought by high-intensity ultrasound played a key role in increasing the probability of successful collisions at the molecular level.

The precursor of formic acid was determined to be 3-deoxyglucosone (3-DG), and 1-deoxyglucosone (1-DG) was the precursor of acetic acid.^34^ Yu et al.^6^ studied a MR model at pH 10.0, and concluded that the alkaline condition would significantly promote the generation of 1-DG, hence, resulting in a high amount of acetic acid and pyrazines generated as final MRPs. The results of melanoidins again proved the promotion of MR after introducing high-intensity ultrasound into the oil-in-water MR systems. Previous studies have proposed two mechanisms on the promotion of MR due to oils. Firstly, after both thermal and ultrasonic processing, oil oxidation became severe and a number of lipid oxidation products were generated, e.g., aldehydes and carbonyls.^35^ These lipid oxidation products were capable of reacting with intermediate MRPs and subsequently contributed to generating an increased amount of final MRPs. Secondly, some final MPRs were lipo-soluble and might further migrated to the oil phase; from chemical kinetic view point, removal of final products from the reaction system was an effective method of accelerating the speed of reaction.^36^ The promotion of generating coloured MRPs in the oil-in-water MR systems may be attributed to the first mechanism since the melanoidins were generally lipophobic. In addition, an acceleration of forming some volatile MRPs might be explained by the second mechanism due to their lipophilicity.

### Analysis of volatile compounds

Judging from the amount of pyrazines, the olive and sunflower oil-in-water MR systems showed the best performance on promoting the MR via Strecker degradation among the four oil-in-water MR systems after ultrasonic processing. Meanwhile, sunflower oil was the cheapest oil among the selected oils in the local market, followed by peanut, olive and flaxseed oils. Thus, in terms of increasing the amount of pyrazines generated through the oil-in-water MR systems and reducing the costs, adding sunflower oil into the MR model might be a preferable choice among the four oils.

There are two key findings derived from detecting flavour compounds. Firstly, it is clear that the four oils had their unique impacts on the formation of pyrazines during processing. Different degree of unsaturation in the oils resulted in different viscosity, and subsequently impacted on the mass transfer between the oil and water phases as well as the partition of the intermediate or final MRPs. Different composition in commercial oils containing other compounds, e.g., antioxidants, pro-oxidants, etc., might also be responsible for causing the differences in flavour profiles. Another key finding in this study was that the amount of pyrazines was significantly increased in the four oil-in-water MR systems after ultrasonic processing compared with the same systems upon thermal processing. As discussed in the previous section, high-intensity ultrasound was able to achieve a better mixing-effect than stirring in thermal MR, which may be one of the key reasons for bringing such a promotion.

## Methods

### Preparation of oil-in-water system

The water phase consists of MR precursors and was prepared by dissolving equal molar (0.1 mol) of both d-glucose (Megachem, Singapore) and glycine (Suntop Enterprise, Singapore) in a container with 1.0 L deionized water. Subsequently, the pH of sample solution was adjusted to 10.0 by adding sodium hydroxide (GCE, Singapore). The four types of edible oil were sunflower oil (Ngo chew Hong Pte. Ltd., Singapore), peanut oil (Knife, Lam Soon Pte. Ltd., Singapore), olive oil (Aceites Borges Pont, S.A.U., Spain) and flaxseed oil (Proteco God Pty. Ltd., Australia). Each type of oil was accurately measured at 250 mL and subsequently added into the water solution container. The ratio of oil and water phases was kept constant at 1:5 for each sample. In order to form a stable system during processing, mixing was conducted by placing the sample container on a hot-plate stirrer (CMAG HS-7, IKA, Malaysia) with agitation at 150 rpm and 30 °C for 5 min.

### High-intensity ultrasonic processing and thermal processing

The setup of both high-intensity ultrasonic and thermal processing system followed those of Yu, et al.^6^ Briefly, a continuous ultrasonic tank reactor (CUTR) was connected to an ultrasonic generator (UIP1000, Hielscher, Germany), which output ultrasound wave with intensity of 11.90 W cm^−2^ and frequency of 20 kHz. The ultrasonic power dissipated into the reaction system was 51.71 W (0.22 W cm^−3^). The distance between an ultrasonic probe (BS2d34 with frontal diameter of 34 mm) and the bottom of the CUTR was 3.2 cm. A peristaltic pump (MU-D01, Major Science, USA) continuously pumped the prepared sample solution from a feed tank to the inner chamber of the CUTR, and the total volume of inner chamber was 235 mL. A refrigerated cooling water circulator (WBL-100, MRC, Israel) kept circulating cooling water into the outer jacket of the CUTR for maintaining a stable processing temperature inside the chamber. In our study, the reaction temperature was maintained at 80 °C, and the residence time was set to 60 min. For each trial, the prepared sample solution was firstly pumped into the inner chamber of the CUTR ahead of ultrasonic processing. It took around 5–8 min for the temperature of sample to reach the desired level based on different residence time and processing temperature conditions, and the processed sample was only collected after the steady-state was achieved. The thermal processing was conducted in the same reactor without the assistance of high-intensity ultrasound. Both ultrasonic and thermal processing lasted for 1 h at 80 °C.

After either ultrasonic or thermal processing, 50 mL of the sample was collected into a centrifuge tube (CELLSTAR, Greiner Bio-one, Germany) upon reaching a designed condition, followed by placing it into an ice water bath for a rapid cooling. The tube was then centrifuged at 10414×*g* for 10 min at 25 °C (5810 R, Eppendorf, Germany). The processed oil phase was subsequently separated and collected for further analysis. The rest of the sample, however, remained to be cloudy and creamy after the separation. Li et al.^37^ reported that an emulsion was normally stabilised by phospholipid and carbohydrates. To destabilise the emulsion, 0.8 g sodium chloride (GCE, Singapore) was added to the 4 mL of cream in a 10 mL glass crew-thread vial and agitated at 150 rpm for 5 min over the hot-plate stirrer (30 °C). A clear water phase was then separated and collected for further analysis.

### Analysis of oil phase

The following analytical methods were carried out to determine oil properties: AOCS official method Cd 3d-63 (AV), AOCS official method Ja 8–87 (PV), AOCS official method Da 15–48 (IV, Wijs method), and AOCS official method Cd 18–90 (*p*-anisidine value).

Fatty acid composition analysis was conducted by a gas chromatography connected to a mass spectrometer and a flame ionisation detector (GC-MS/FID). A pre-column derivatization of oils was conducted in order to convert fatty acids to its corresponding fatty acid methyl esters (FAMEs) with increased volatility. Raw oils and oil phase of the processed samples were first saponified and subsequently methylated. Briefly, two drips of the oils were collected and accurately weighted and subsequently transferred to a 15-mL reaction tube with screw cap (Sterilin, ThermoFisher Scientific). One and a half mL of 0.5 mol L^−1^ sodium hydroxide–methanol solution was added into the tube and blanked with N_2_ gas followed by heating for 5 min in a boiled water bath (TW12, Julabo, Germany). After cooling down to room temperature, 2 mL of 14% (w/w) boron trifluoride in methanol solution (Sigma Aldrich, USA) was added as a derivatizing agent, mixed thoroughly, and then heated for 30 min in the boiled water bath. An internal standard, methyl tricosanoate (≥99%, Sigma Aldrich, USA), was accurately weighted (0.14 g) and completely dissolved into 25 mL of hexane. One and a half mL of methyl tricosanoate-hexane solution was added to the reaction tube after cooling down to 30–40 °C. The tube was blanketed by N_2_ gas, capped, and shook vigorously for 30 s on a vortex mixer (Vortex-Genie 2, Scientific Industries, USA) when it was still warm. Subsequently, 5 mL of saturated sodium chloride solution was added into the tube in order to cease the reaction. The tube was agitated thoroughly and rested until achieving a well separation. The top (hexane) layer was carefully transferred to a cleaned sample vial (1.5 mL) and then blanketed by N_2_ gas.

The GC-MS/FID system (QP2010 Ultra, Shimadzu, Japan) was coupled with a BPX70 polar column having 120 m × 0.25 mm I.D. and film thickness of 0.25 μm (SGE Analytical Science, USA) for the FAMEs analysis. The injection volume was set to 1 μL, and the split ratio was 1:50. The carrier gas was helium with a constant flow rate of 1.00 mL min^−1^. The temperature programme was set as follows: the initial oven temperature was set to 170 °C followed by a linear increase to 225 °C at 2 °C min^−1^ with a hold time of 30 min. The oven temperature was subsequently increased to 250 °C at a rate of 20 °C min^−1^ and then remained constant for 2 min. The total programme time was 60.75 min. Each FAME peak was firstly identified by the MS and then quantified corroding to its corresponding peak area in the FID result.

### Analysis of water phase

Quantification of d-glucose, fructose, formic and acetic acids was performed by high-performance liquid chromatography (HPLC) analysis. The HPLC system consisted of a Supelco C-610H column (300 mm × 8 mm, Sigma-Aldrich, USA) and tandem detectors of refractive index detector (sensitivity: 64, temperature: 30 °C) and photodiode array detector (wavelength at 210 nm) (Waters, USA). Mobile phase was 0.1% phosphoric acid-deionized water solution, and column temperature was set at 30 °C. The analysis lasted for 30 min.

For quantification of glycine in the water phase, glycine was firstly derivatized by the AccQ Fluor reagent and subsequently injected into a HPLC system including an AccQ-Tag, 3.9 × 150 mm column and a photodiode array detector (2414, Waters, USA). The injection volume, column temperature and detection wavelength were set to 10 μL, 37 °C and 254 nm, respectively. Mobile phase A was prepared by diluting the concentrated AccQ-Tag Ultra Eluent A with deionized water at a ratio of 1:10. Mobile phase B was 60% v/v acetonitrile-water solution. The HPLC gradient programme was the same as described in Yu et al.^6^

Quantification of melanoidins in water phase was conducted by measuring the absorbance at 470 nm on an ultra-violet-visible spectrophotometer (UV-vis 1280, Shimadzu, Japan). The concentration of melanoidins was obtained based on the principle of Lambert-Beer with an extinction coefficient of 0.64 ± 0.03 L mmol^−1^ cm^−1^ (ref. ^3^).

### Analysis of flavour compounds

Flavour compounds in raw oils and processed samples (both oil and water phases) were extracted using a solid phase micro-extraction fibre and then injected into the GCMS for further analysis. Both procedures of extraction and GCMS analysis in this study were the same as described in Yu et al.^7^

### Statistical analysis

All experiments were conducted in triplicate. All experimental data were subject to the analysis of variance (ANOVA) with Duncan’s multiple range test using the SPSS statistical software (version 22, IBM, USA) for detecting significant differences (*p* < 0.05). The values and error bars in figures and tables are reported as mean value and standard deviation, respectively.

## Conclusions

This study firstly reported the effects of high-intensity ultrasound on a d-glucose and glycine MR in oil-in-water systems and analysed both oil and water phases regarding changes of oil properties, reactants of MR and MRPs. Furthermore, a comparison between ultrasonic and thermal MR was made and subsequently some possible mechanisms were proposed. Major advantages of introducing high-intensity ultrasound into the model system included accelerating the reaction rate of MR and generating a significantly high amount of desired flavour compounds. A significantly high, albeit momentary, temperature and pressure condition generated by high-intensity ultrasound was suggested to be the main reason for bringing about such a promotion of MR. In the oil-in-water MR systems, a well-mixing effect brought by high-intensity ultrasound was another important factor for increasing the rate of MR. On the other hand, some drawbacks included an increased degree of oxidation in the ultrasound-processed oils compared with that in the raw oils, which decreased the amount of nutrients in oils and released off-flavours simultaneously.

### Data availability

The datasets generated during and/or analysed during the current study are available from the corresponding author on reasonable request.
